# Flow Cytometric Findings in Primary Effusion Lymphoma: A Report of Two Cases

**DOI:** 10.7759/cureus.25637

**Published:** 2022-06-03

**Authors:** Ahmad Alkhasawneh, Khaled S Mohamed, Ketav Desai, Reeba Omman, Brett Baskovich

**Affiliations:** 1 Pathology, University of Florida College of Medicine, Jacksonville, USA; 2 Pathology and Laboratory Medicine, University of Florida College of Medicine, Jacksonville, USA

**Keywords:** ebv, human herpesvirus-8, flow cytometry, effusion, lymphoma

## Abstract

Primary effusion lymphoma (PEL) is a rare B-cell lymphoma that usually occurs in the setting of HIV infection, and it is associated with Human Herpesvirus-8 (HHV-8). Diagnosis of PEL is usually established in cell centrifuge, cell block, or tissue examination, and there are few reports describing flow cytometry findings in PEL. We report two male patients (a 34-year-old and a 56-year-old) with a history of HIV infection. The first patient presented with ascites and abdominal pain, and the second patient presented with chest pain and parapneumonic pleural effusion. Cavitary fluid examination showed large pleomorphic neoplastic lymphoid cells with plasmablastic morphology. Flow cytometry analysis of the neoplastic lymphocytes showed increased forward scatter and side scatter with intermediate to a high level of CD38 expression. In one patient, lymphoma cells showed bright CD45 expression with dim expression of CD19 and kappa light chain. There was no significant expression of CD20 or any T/NK cell markers in either case. Immunohistochemistry for CD30 was positive in one patient. Immunohistochemistry for HHV-8 and in situ hybridization for Epstein-Barr virus-encoded small RNAs (EBER) was positive on cell blocks in both cases, consistent with the diagnosis of primary effusion lymphoma. PEL should be considered in the differential diagnosis of CD20-negative hematopoietic neoplasms, and flow cytometry may provide helpful clues for the diagnosis of PEL as part of the workup for pleural effusion with cytologically malignant cells.

## Introduction

Primary effusion lymphoma (PEL) is a rare aggressive type of B-cell lymphoma that is universally associated with Human Herpesvirus-8 (HHV-8), and it commonly occurs in the setting of HIV-infected male patients [1,2]. PEL patients most commonly present with cavitary malignant effusion such as pleural, pericardial, or peritoneal effusion, and less often as extracavitary mass or both [2,3]. On cytologic or histologic examination, the tumor cells are large and pleomorphic, and they exhibit plasmablastic, immunoblastic, or anaplastic morphology. The diagnosis of PEL is established by combining morphologic findings with supporting immunohistochemical profiles, including reactivity for HHV-8 with or without concurrent Epstein-Barr virus (EBV) infection. PELs usually express activation markers such as CD30 and CD38, and there is often a lack of pan B-cell marker expression, including CD19, CD20, and CD79a.

Flow cytometry is a powerful diagnostic tool in the daily practice of hematopathology, especially in the evaluation of the presence of chronic lymphoproliferative disorders and acute leukemias, and it has the advantage of providing quantitative analysis for the expression of markers within a short turnaround time. However, flow cytometry may not be routinely performed on certain body fluid specimens in some institutions, even though the diagnosis is still reliable in the evaluation of cell centrifuge or tissue specimens. In addition, hematopathologists may not be aware of concerning findings in the cellblock or the clinical information when the flow cytometry evaluation is performed at a reference laboratory.

Very few studies have described the flow cytometric phenotype of primary effusion lymphoma [2,4], and herein we report the flow cytometry findings in two cases of PEL performed as a part of the workup for cavitary fluid at our institution.

## Case presentation

We describe two cases of primary effusion lymphoma with emphasis on flow cytometry findings.

Case 1

**Figure 1:**
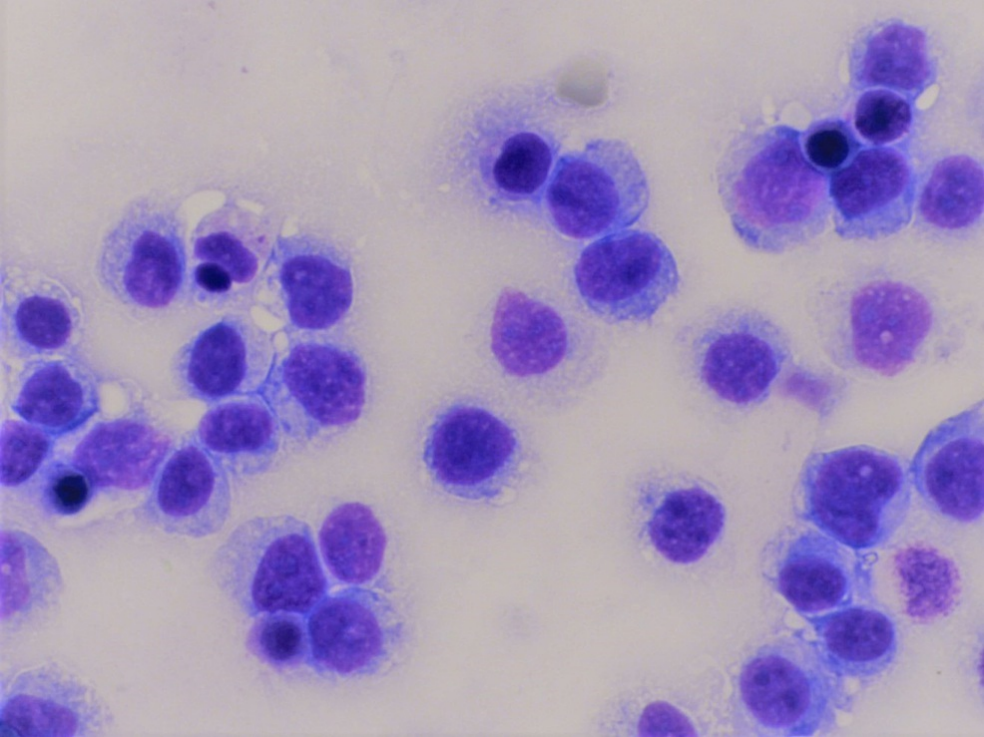
Primary effusion lymphoma (case 1) The Giemsa stain shows large neoplastic lymphocytes with a moderate amount of cytoplasm consistent with plasmablastic morphology (630x).

The first case is of a 36-year-old man with a past medical history of HIV who presented with abdominal pain and ascites in July 2017. At the time of presentation, his cluster of differentiation 4 (CD4) count was 132.2 cells/microliter. Examination of the ascitic fluid showed large malignant pleomorphic lymphocytes with plasmacytoid morphology consistent with plasmablastic morphology, and some showed prominent nucleoli (Figure 1).

**Figure 2:**
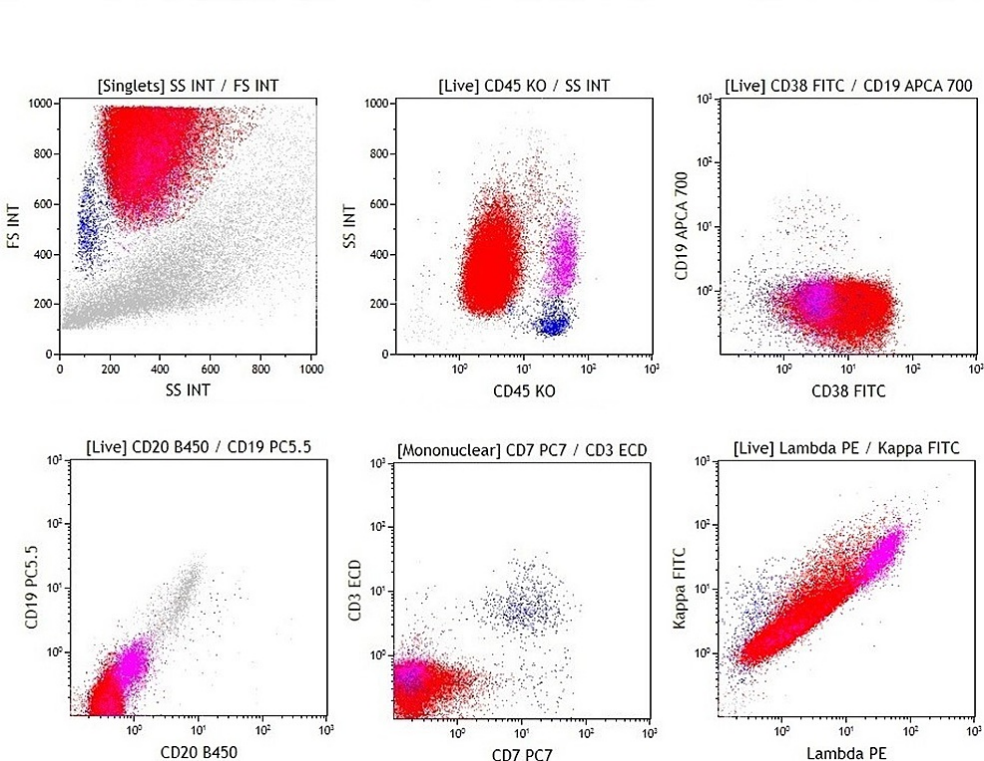
Primary effusion lymphoma (case 1) Dot plots show an abnormal population (red population) with increased forward scatter and side scatter. The neoplastic lymphocytes are positive for CD38, and there is no significant expression of CD45, CD19, CD20, CD3, CD7, or surface light chains.

Flow cytometry showed an abnormal CD45-negative population with increased forward scatter and side scatter with variable expression of CD38 (Figure 2, Table 1).

**Table 1:** Immunophenotype of primary effusion lymphoma with the intensity of expression (0: negative, 1: dim, 2: intermediate, 3: strong). * T/NK cell markers included CD2, CD3, CD4, CD5, CD7, CD8, and CD56.

Flow cytometry marker	Case 1	Case 2
T / NK markers*	0	0
CD10	0	0
CD11c	0	0
CD19	0	0-2
CD20	0	0
CD34	0	0
CD38	0-2	2-3
CD45	0	2-3
CD138	0	0-2
Surface kappa	0	1
Surface Lambda	0	0
Cytoplasmic kappa	0	1
Cytoplasmic lambda	0	0
Fluorescence intensities are 0 to 3.		

There was no significant expression of CD19, CD20, surface light chains, or T/NK cell markers by flow cytometry. Immunohistochemistry showed that the neoplastic cells were positive for HHV-8, and there was no expression of CD3, CD20, or CD30. In situ hybridization for EBER was positive. Flow cytometric CD138 and cytoplasmic light chain analysis were performed at a reference laboratory on a recurrence sample, and lymphoma cells did not express these markers.

Staging bone marrow showed no evidence of involvement by lymphoma. The patient was in remission for 10 months after the first line of chemotherapy treatment at another institution, and he developed recurrence presenting as ascites and small bowel obstruction due to involvement by PEL in early 2019. His second-line chemotherapy treatment was not successful, and he expired after nine months of recurrence.

Case 2

The second case is of a 56-year-old man with a past medical history of untreated hepatitis C virus and HIV/AIDS who presented with dyspnea, chest pain, fever, night sweats, and cough in October 2020. At presentation, his CD4 count was 128 cells/microliter, and the HIV viral load was 177k copies/mL. He was initially treated empirically for multilobar pneumonia with bilateral parapneumonic effusion. His blood culture grew non-lactose-fermenting gram-negative rods, and cultures from the pleural effusion fluid were negative for bacteria, fungi, and mycobacteria. He was switched to broad-spectrum antibiotics, and his fever resolved; however, his dyspnea and pleural effusion persisted.

Pleural fluid cytology showed atypical large neoplastic lymphocytes with a moderate amount of cytoplasm.

**Figure 3:**
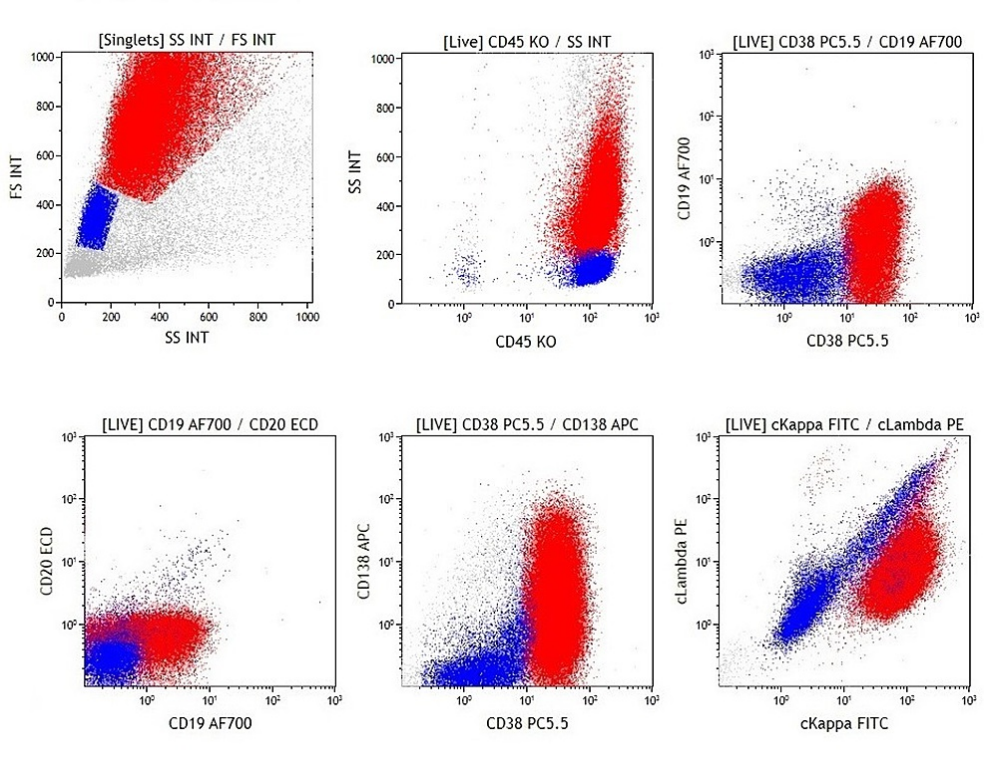
Primary effusion lymphoma (case 2). Dot plots show a neoplastic population with high forward scatter and side scatter (red: abnormal population, blue: normal cells). The neoplastic lymphocytes are positive for CD45, CD38, and cytoplasmic kappa, and there is variable expression of CD19 and CD138. CD20 is negative.

Flow cytometry showed an atypical CD45-positive population of monoclonal cells with increased side scatter, CD38 expression, and variable CD19 and CD138 (Figure 3, Table 1).

Immunohistochemistry performed on the cell block showed reactivity for HHV-8 and CD30 in neoplastic lymphocytes, and in situ hybridization for EBER was positive. The overall findings were consistent with primary effusion lymphoma.

The patient’s staging bone marrow examination was negative for involvement by lymphoma. CT imaging of the chest, abdomen, and pelvis showed diffuse lymphadenopathy, mostly subcentimeter, but there were bilateral enlarged inguinal lymph nodes (2 cm). Pleurodesis was performed during his initial admission and in March 2021, and there was no clinical recurrence on his last imaging and follow-up last March after chemotherapy treatment.

## Discussion

PEL is a rare B-cell malignancy that is universally associated with HHV-8 infection, and approximately two-thirds of cases are associated with EBV infection [1,2]. PEL most commonly occurs in HIV-positive male patients, though it may occur in HIV-negative patients and the posttransplant setting [5]. While it was originally described as body-cavity-based lymphoma [6], cases with solid tumor masses (so-called extracavitary variant PEL) have been described in the gastrointestinal tract, skin, and central nervous system [2,7]. Both cavitary and extracavitary PEL are recognized under PEL in the WHO classification [1].

PEL typically shows plasmablastic, immunoblastic, or anaplastic morphology. Generally, PEL lacks the expression of lineage-specific markers (null-cell phenotype), including pan-B-cell antigens (CD19, CD20, and PAX5) and T/NK cell antigens. Gene rearrangement studies show immunoglobulin gene rearrangement [8]. The neoplastic lymphocytes are generally reactive to CD45 and multiple myeloma 1 (MUM1) and express activation markers such as CD30 and CD38. In addition, light chain restriction can be identified in less than 50% of cases, and CD3 can be expressed in up to 20% of cases [2]. Diagnosis of PEL is usually established by a review of cytomorphology combined with supporting ancillary studies such as immunohistochemistry for HHV-8 and in situ hybridization for EBER.

We described the flow cytometric characteristics of two cases of PEL in association with cavitary effusion. The neoplastic cells showed increased forward scatter and side scatter, and CD38 was expressed in both cases. One case showed the null phenotype, and the other showed a strong expression of CD45 with a partial expression of CD19 and CD138.

There is a significant clinical, morphologic and phenotypic overlap between PEL and other hematopoietic neoplasms, and the distinction can be difficult, especially in the setting of extracavitary lesions. The differential diagnosis of PEL includes plasmablastic lymphoma, plasmablastic myeloma/plasmacytoma, anaplastic large cell lymphoma, HHV-8 positive large B-cell lymphoma, and other CD20-negative large B-cell lymphomas. Flow cytometry often shows similar side scatter properties (forward scatter and side scatter) in these lymphomas, and careful evaluation for other markers combined with additional studies for anaplastic lymphoma kinase (ALK), HHV-8, and EBER plays a major role in establishing the correct diagnosis. Poorly differentiated carcinoma and melanoma may show clinical and morphologic overlap with PEL, and additional studies to evaluate for these malignancies might be necessary as well.

Plasmablastic lymphoma (PBL) also occurs predominantly in HIV-infected male patients, and EBV infection is present in around two-thirds of PBL cases [9]. The morphology of PBL and plasmablastic myeloma (PBM) will be similar to PEL, and they may show the same phenotype (i.e., expressing plasma cell or activation markers with no expression of CD19 or CD20) [10]. Flow cytometry may provide clues in distinguishing PBL or plasmacytoma from PEL as CD45 is generally negative in PBL and PBM, and the presence of cytoplasmic light chains restriction can be demonstrated in most cases of PBL and PBM [9]. Immunohistochemistry for HHV-8 is most useful, as HHV-8 is not expressed in PBL or PBM. In addition, myeloma is a bone marrow disease with osteolytic lesions in many cases, though PEL may rarely involve bone marrow at presentation [2].

Anaplastic large cell lymphoma (ALCL) is a T-cell lymphoma with pleomorphic and anaplastic morphology with a strong expression of CD30. It is divided into ALK-positive ALCL and ALK-negative ALCL based on ALK status. The morphology of ALCL resembles PEL and PBL, and CD3 and CD30 can be present in PEL and PBL as well [11]. Flow cytometry may provide clues indicative of ALCL. For example, ALCL is very often positive for CD45, and there will be an expression of one or more T-cell markers, such as CD4 and CD2 [12]. ALCL does not express light chains or pan-B-cell markers (CD19/CD20) [12-14]. The presence of EBER or HHV-8 argues against the possibility of ALCL, and the expression of ALK is not compatible with PEL. 

ALK-positive large B cell lymphoma (ALBCL) is another PEL-like lymphoma that shows immunoblastic morphology and a plasma cell immunophenotype [1,15,16]. Similar to PEL, immunophenotyping of ALBCL shows an expression of plasma cell markers with a lack of pan-B-cell antigens [15]. ALBCL often expresses CD45 and shows light chain restriction. Evaluation for HHV-8, EBER, and ALK is most helpful in this distinction, and ALK is positive in ALBCL, while HHV-8 and EBER are negative. HHV-8 positive large B cell lymphoma is yet another PEL-like lymphoma that usually occurs in the setting of HHV- 8 positive multicentric Castleman’s disease [17] and has the same morphology as PEL. Flow cytometry shows a similar phenotype, but the presence of EBER supports PEL.

Poorly differentiated carcinoma and melanoma often show a CD45-negative population with similar side scatter properties to PEL and may mimic PEL on a limited flow cytometry panel. Flow cytometric positivity for BerEP4 is consistent with an epithelial neoplasm [18]. In addition, there is no expression of HHV-8 in carcinoma or melanoma, and the expression of other epithelial markers or melanocytic markers can be helpful in the workup. 

## Conclusions

Correlating the specimen type with clinical and morphologic findings is most useful in formulating the differential diagnosis, especially for a cavitary effusion. Flow cytometry may suggest the presence of PEL or PEL-like lymphoma, which would guide pathology work up towards hematopoietic neoplasm, especially in specimens with low cellularity. Flow cytometry findings in PEL include increased light scatter, null phenotype, and expression of plasma cell markers (CD38). The presence of these finding should prompt further work up, including evaluation for CD30, HHV-8, EBER, ALK and light chain assessment with or without staining for cytokeratin and S100. The presence of surface and/or cytoplasmic light chain restriction would indicate a B-cell or plasmablastic neoplasm. The expression of CD45 along with CD30 and more than one T-cell marker provides clues suggesting other entities such as ALCL.

In conclusion, flow cytometry may provide helpful clues for PEL, which should be included in the differential diagnosis of a CD20-negative lymphoid malignancy with expression of plasma cell markers.
